# Inflammatory Cytokines Associated with Obesity, Type-2 Diabetes, and Hypertension Exacerbate Breast Cancer Risk in Underserved African American and Latin American Women

**DOI:** 10.3390/jcm13061687

**Published:** 2024-03-15

**Authors:** Yanyuan Wu, Eduard Karapetyan, Pranabananda Dutta, Magda Shaheen, Jaydutt V. Vadgama

**Affiliations:** 1 Division of Cancer Research and Training, Department of Medicine, Charles R. Drew University of Medicine and Science, Los Angeles, CA 90059, USA; ekarapetyan92@gmail.com (E.K.); pranabandutta@cdrewu.edu (P.D.); magdashaheen@cdrewu.edu (M.S.); 2 Jonsson Comprehensive Cancer Center, David Geffen School of Medicine, University of California at Los Angeles, Los Angeles, CA 90095, USA

**Keywords:** comorbidity, breast cancer, cytokines, African American, Latin American

## Abstract

**Background:** Comorbid chronic diseases, such as obesity, Type-2 Diabetes (T2D), and hypertension (HTN), are major public health issues and highly prevalent among underserved African Americans (AA) and Latin Americans (LA). Elevated inflammatory cytokines are underlying processes in comorbidities (obesity, T2D, and HTN) that could contribute to tumorigenesis and adverse cancer outcomes. **Methods:** A panel of 19 cytokines was measured by Luminex assay from 570 AA and LA women’s serum samples. The comorbidities and breast cancer information were extracted from our existing clinical database. Comorbidity-associated cytokines were identified by linear regression analysis, and the odds ratios of increasing cytokines for breast cancer were evaluated by Logistic regression. **Results:** Women with obesity, T2D, and HTN elevated specific groups of cytokines. EGF, MCP1, MDC, MIP-1b, and Groα were independent of T2D and HTN significantly associated with obesity. TGFβ1 and TGFβ2 were T2D-associated cytokines, and MIB-1b, TNFα, and VEGFα were HTN-associated cytokines. Among those comorbidity-associated cytokines, CXCL1, CCL4, CXCL10, TNFα, TGFβ1, and TGFβ2 were also significantly associated with breast cancer diagnosed at age < 50. Two or more comorbidities further increased the levels of Groα, MIP-1b, TNFα, and TGFβs. **Conclusions:** Comorbidity-associate cytokines could augment the risk of breast cancer for AA and LA women.

## 1. Introduction

Obesity has been a significant health challenge in the U.S. and globally. According to data from the Centers for Disease Control and Prevention (CDC), non-Hispanic Black adults (49.9%) had the highest age-adjusted prevalence of obesity, followed by Hispanic adults (45.6%), non-Hispanic White adults (41.4%), and non-Hispanic Asian adults (16.1%) [1]. Individuals with obesity have an increased risk of cancers, such as colon, prostate, and postmenopausal breast cancer, and are associated with high cancer mortality rates [2,3,4]. Our previous study also identified an association between obesity and breast cancer in postmenopausal African American women [5]. African American women with obesity or body mass index (BMI) > 28 significantly reduced disease-free survival [5]. 

Individuals with obesity are at risk of developing metabolic syndrome, type-2 diabetes (T2D), and hypertension (HTN) [6,7]. An umbrella study shows that breast, endometrial, and colorectal cancer incidence was more significant in individuals with type-2 diabetes than in those without diabetes [8,9]. Hypertension is also a known risk factor for renal cancer in both men and women [10]. It was reported that hypertension increased the risk of renal cancer by two-fold in Caucasian and three-fold in African American patients [11]. Furthermore, hypertensive men are at a higher risk of developing prostate cancer, and hypertensive women are at a higher risk of developing endometrial and breast cancers [12]. A meta-analysis of 30 published studies with 11,643 breast cancer cases showed that postmenopausal women with hypertension might have a 15% increased risk of breast cancer [13]. Additionally, hypertension patients may increase cancer mortality risk by 7–15% compared with normotensive patients [14,15]. Comparing different ethnic groups, African Americans (AA) and Latin Americans (LA) have a high prevalence of type-2 diabetes [16]. Hypertension is also more common in AA [17]. Elevated rates of comorbidities of obesity, T2D, and/or HTN were also often seen in AA and LA [18]. The combined effects of comorbidities increase the risk of cancer incidence [19]. 

The molecular mechanisms underlying the risk of cancers with comorbidities are complex. Potential mechanisms linking obesity, T2D, and cancer include metabolic conditions such as hyperinsulinemia and dyslipidemia and the alteration of adipose tissue characterized by inflammation and a tumor growth-promoting secretory profile [8,20]. Obesity, especially abdominal obesity, increases adipose tissue inflammation with the production of cytokines and changes in the circulating concentrations of adipokines [21]. The circulating cytokines promote tumor angiogenesis, stimulate the cancer stem cell population, drive cancer growth, and promote tumor invasion and metastasis [22]. The effects of obesity on the risk of breast cancer in premenopausal and postmenopausal women also differ [20,21,22]. Overall, cancer and HTN may share common risk factors. Furthermore, breast cancer and HTN may share a common pathophysiological pathway mediated by adipose tissue, which could cause chronic inflammation and further increase the risk of breast cancer and HTN [23,24]. Many pathways that may be altered in HTN are also associated with neoplastic growth [25]. Notably, comorbidities associated with chronic inflammation-stimulating cytokines could be an important mechanism underlying the comorbidities and cancer relationship. 

Cytokine profiles related to obesity, T2D, and HTN were studied in different populations [26,27,28,29]. Several studies were focused on AA with obesity, T2D, and HTN [27,28,29]. Williams et al. used a case-matched approach in their research and identified cytokines that may contribute to the development and onset of T2D in obese AA women [27]. Denis’s study included 39 obese AA women and was designed to identify cytokine associations with BMI and T2D [28]. DeLoach’s study recruited 484 young AAs from the local community with and without obesity and/or HTN only. A strong association between BMI and inflammation cytokines was identified. However, the study examined only five cytokines [29]. None of those studies evaluated inflammation cytokines for the risk of breast cancer in AA with obesity, T2D, and HTN. 

Cytokine profiles related to different cancers and cancer progression were studied in cancer patients [30,31,32,33,34]. These studies were focused on identifying circulating cytokine as biomarkers for the early detection of breast cancer [30], characterization of breast cancers [31], and better assessing cancer progression [32,33,34]. None of those studies focused on the AA and LA populations. There is a lack of information to evaluate the cytokine profiles associated with comorbidities related to cancer risks in the same cohort. Specifically, there is a lack of data on cytokine profiles in LA, who also suffer from high comorbidities. The AA and LA communities in South Los Angeles suffer from disadvantaged neighborhoods and experience significantly more comorbidity and high incidences and mortality rates of breast cancer. The disadvantaged social determinants of health and comorbidities could increase inflammatory cytokines and influence breast cancer incidence and survival in AA and LA women, contributing to health disparities. Therefore, employing biomarkers, such as cytokines, and prioritizing strategies reducing inflammatory cytokines could improve women’s health outcomes. Our study’s uniqueness lies in it (1) having a relatively large sample size that includes AA and LA women with similar socioeconomic status and neighborhood environmental factors from the local community, which allows the identification of specific cytokines markers associated with AA or LA; (2) considering the most common comorbidities, obesity, T2D, and HTN existing in AA and LA communities; (3) profiling serum cytokines levels combined with comorbidities that could provide a more personalized risk assessment for breast cancer in AA and LA women with comorbidities. The identified panel of cytokines could serve as an intermediate outcome marker in prevention studies for the AA and LA communities. The panel of cytokines makers identified from this study and other known biomarkers could also be used to manage breast cancer patients better.

## 2. Materials and Methods

### 2.1. Human Subjects

**Figure 1:**
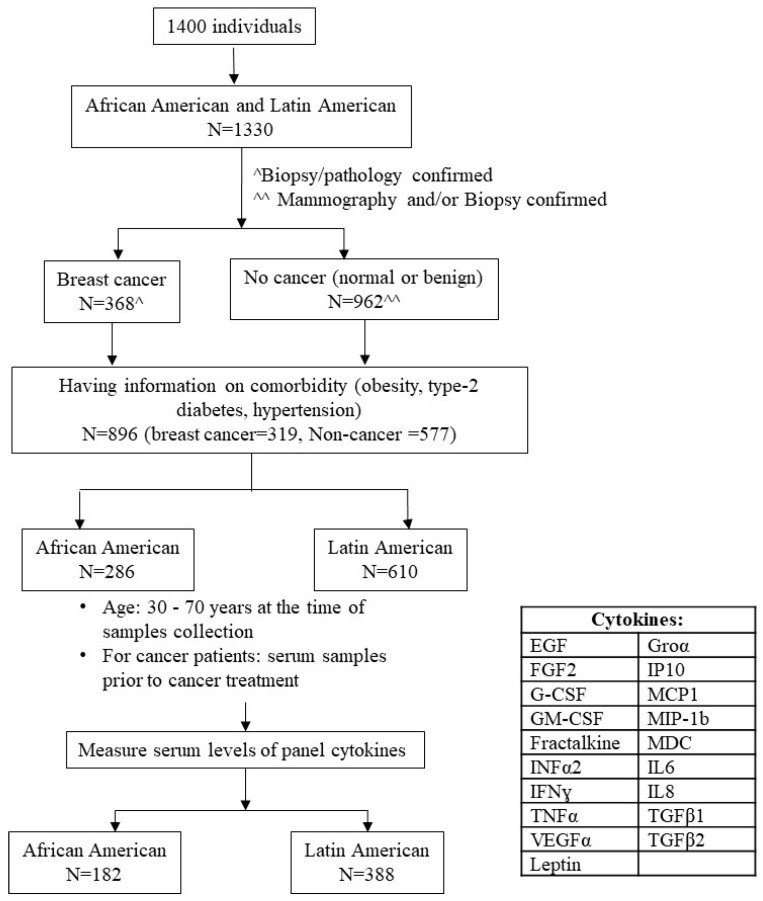
Subjects’ selection. The flowchart demonstrated subjects’ selection process for the study. ^ Breast Cancer cases (*n* = 368) were confirmed by biopsy and having documented pathology report. ^^ No Cancer individuals (*n* = 962) were confirmed by either mammography as normal or by biopsy as benign.

The study was approved by the University’s Institutional Review Board (# IRB 00-06-041). The study population was recruited from the Service Planning Area 6 (SPA6) region of South Los Angeles County in California. The population by race/ethnicity in SPA6 is 28% AA, 68% LA, and 4% other, including Caucasian, Asian, Native American, and Pacific Islander. The cohort comprised women examined in the Mammography Clinic or the Hematology/Oncology Clinic at the Martin Luther King Ambulatory Care Center (MACC, formerly known as King-Drew Medical Center) between 1998 and 2019. Women consented to an ongoing breast cancer study in the Division of Cancer Research and Training at Charles R. Drew University of Medicine and Science and MACC. At the time of recruitment, study coordinators conducted survives for each consented individual regarding their family history, socioeconomic status, and personal life-related risk of breast cancer and collected their blood samples as baseline samples. Study coordinators also collected individuals’ health conditions, such as comorbidities, from their medical records. For follow-up data, we conducted post hoc medical record abstraction. For women with breast cancer, the follow-up was performed along with each cancer treatment protocol first. Once cancer-free, the follow-up was continued yearly for 5 years. Blood samples were collected during the follow-up, and clinical information, including medication, cancer conditions, and other health information, was documented. Women without breast cancer were followed up yearly for 5 years. Figure 1 illustrates the process of selecting the subset of individuals for this study from a total number of women (*n* = 1400). 

The inclusion/exclusion criteria were as follows. (a) Self-identified race/ethnicity: 31.9% were AA, and 68.1% were LA. Considering that the number of Caucasian and Asian participants was small and may not have generated meaningful statistical analysis, we only included AA and LA (Hispanic/Latin women) in this study. (b) Having information on comorbidity, i.e., obesity, T2D, and/or HTN. The comorbidity occurred before breast cancer diagnosis for women having breast cancer. (c) Breast cancer status was confirmed by the biopsy/pathology of the breast tissue, and only subjects who had documentation of this information were included in the study. Non-cancer controls included healthy women who came for routine mammogram checking, had breast lumps, and ruled out malignancy. (d) Baseline blood sample (serum sample collected at the time of diagnosis and before cancer treatment for breast cancer). We selected (e) those aged 30–70 years at the time of blood sample collection to assess levels of panel cytokines. A total of 570 women who fulfilled our inclusion criteria and their serum level of panel cytokines were obtained successfully. 

### 2.2. Demographic and Clinical Information Collection

Ethnicity was determined from self-reports at the time of recruitment. Body mass index (BMI) is <18.5 Underweight, 18.5–24.9 Normal Weight, 25–29.9 Overweight, ≥30 Obese. It should be noted that no Underweight women (BMI < 18.5) were identified in the study cohort. Thus, this category is omitted from analyses. T2D was diagnosed as a glycated hemoglobin (A1C) level ≥ 6.5% or fasting blood sugar level ≥ 26 mg/dL (7 mmol/L) on two separate tests. HTN was diagnosed when blood pressure was consistently ≥130 and/or ≥80 mm Hg. The biopsy/pathology of the breast tissue confirmed the breast cancer diagnosis. All that information had documentation included in the study.

### 2.3. Cytokines Penal and Measurement

The MILLIPLEX MAP 15 human cytokine/chemokine panel, EGF, FGF2, G-CSF, GM-CSF, Fractalkine (CX3CL1), INFα2, IFNɣ, TNFα, IP10 (CXCL10), MCP1(CCL2), MIP-1b (CCL4), MDC (CCL22), IL6, IL8, and VEGFα (Cat# HCYTOMAG-60K-15C), were custom made by EMD Millipore, CA, USA. Magnetic beads kits for TGFβ1, TGFβ2 (Cat# TGFBMAG-64K), and Leptin (Cat# HMHEMAG34K) were also made by EMD Millipore, CA, USA. The serum levels of cytokines were determined by Luminex multiplex assay on a Luminex 200 instrument (Luminex, Austin, TX, USA) according to the manufacturer’s recommendations. Briefly, 25 µL of serum per well was incubated overnight with cytokine-specific magnetic beads at 4 °C. The following day, the samples were washed and incubated with 50 µL of detection beads at room temperature (R.T.). Both incubations were performed on a plate-shaker at 800 rpm. Cytokine detection was performed on a Luminex 200 using 100 µL of xMAP™ Sheath Fluid (Cat no. 4050015, Thermo Fisher Scientific, Carlsbad, CA, USA). Data were analyzed using MILLIPLEX™ Analyst v5.1 (Virgene Tech, Carlisle, MA, USA). Each sample was analyzed in duplicate. The serum level of Groα (CXCL1) was measured by the Human CXCL1/Groα quantikine ELISA Kit (Cat# DGR00B, R&D systems, MN, USA) according to the manufacturer’s instruction. Briefly, 200 μL serum samples were added to each well of the Groα-specific ELISA strip and incubated for 1.5 h at R.T. The wells were washed and re-incubated with Groα conjugate for an additional 1 h at 4 °C. In the end, the wells were incubated with the substrate solution for 15 min at R.T. Then, the reaction was stopped by adding a stop solution. The absorbance was read colorimetrically (Promega Glomax Multidetection System, Promega Corporation, Madison, WI, USA) at 450 nm. A standard curve was generated to calibrate the concentration of GROα in each supernatant sample and expressed as the concentration of Groα (pg/mL). The samples were run in duplicates, and the experiment was repeated thrice for consistent results.

### 2.4. Statistical Analysis

Statistical analysis was performed using SPSS software (IBM SPSS Statistics version 22, IBM, Armonk, NY, USA). The normality of the distribution of each cytokine level was evaluated. The Shapiro–Wilk test showed a significant departure of all cytokines from normality. Hence, the levels of cytokines were presented as the median level in this study. Mann–Whitney U (2 samples) or Kruskal–Wallis one-way ANOVA (k samples) tests determine the statistical differences in median levels of cytokines between different ethnicities or among comorbidity conditions. Due to the skewed distribution of cytokines in the study population, the data would not be suitable for analysis with linear regression statistical methods. A logarithm transformation (log base 10) of cytokines’ data was made to ensure these respective data distributions were approximate. A linear regression model estimated the association of each cytokine with obesity, T2D, and HTN adjusted for age, ethnicity, and breast cancer. The Log 10 base cytokine level was used as the dependent variable, and comorbidity conditions were independent variables in the model. After fitting the models, the regression parameter estimates for the log-transformed dependent variables were anti-log transformed to the original scale and presented as geometric mean ratios. Model results were presented as coefficients of obesity, T2D, or HTN and the geometric mean ratios with 95% confidence intervals (C.I.s). To assess the association of each cytokine with breast cancer, the levels of cytokines were stratified according to their percentile ranges, i.e., ≤50 percentile, >50 and ≤75 percentiles, and ≥75 percentile, respectively. The respective percentile levels of cytokines were used as cut-off values for assessing the odds ratio of breast cancer by Logistic regression adjusted for obesity, T2D, HTN, and ethnic groups in age ≤ 50 years and age > 50 years, respectively. The *p* < 0.05 was considered statistical significance in all analyses.

## 3. Results

### 3.1. Study Population and Serum Cytokines Levels

As shown in Table 1, the study population included 570 AA women and AA women aged 30–70 years. Around 32% were AA women, and 68% were LA women. Breast cancer patients made up 45.8%, and 54.2% of women were non-cancerous. According to BMI, 45.8% of women were obese, and 36.8% of them were overweight in this cohort of women. Among the 570 women, 28.1% had T2D, and 33.7% had HTN (Table 1). Around 16% of women had both T2D and HTN. 

Serum levels of the 19 cytokines were measured in the 570 AA and LA women. Table 2 shows cytokines’ median serum levels according to ethnicity, BMI, T2D, and HTN groups. As shown in Table 2, serum levels of EGF, Groα, MIP-1b, MDC, and VEGFα levels were significantly higher in AA women than in LA women. Obesity was associated with higher serum levels of EGF, Groα, MCP1, MIP-1b, MDC, VEGFα, and Leptin. Serum levels of TNFα, MCP1, TGFβ1, TGFβ2, and Leptin were increased in women with T2D, and levels of TNFα, Groα, MIP-1b, MDC, IL8, and VEGFα were higher in women with HTN. Serum levels of EGF, G-CSF, TNFα, Groα, IP10, MIP-1b, MDC, VEGFα, TGFβ1, and TGFβ2 were also high in women with breast cancer compared to the control group (no cancers). The serum cytokine levels were regulated by more than one comorbid condition and may be associated with specific ethnic groups. Further statistical analysis was performed to determine whether each cytokine had an independent association with obesity, T2D, HTN, and breast cancer. 

### 3.2. Identifying Independent Association of Cytokines with Comorbidities in AA and LA Women

A linear regression model adjusted for age, smoking, and alcohol consumption was used to examine the association of obesity, T2D, HTN, breast cancer, and ethnic groups with each cytokine. Table 3 displays the estimated association of obesity, T2D, HTN, and ethnicity on the various cytokines. Due to the skewed distribution of cytokines’ levels, the data were log10 transformed and then included as dependent variables (outcome) in the model. Obesity, T2D, HTN, and ethnicity were all included in the model as covariables to adjust cross-interaction for cytokines. We also included those variables in the model to change the potential influences of age, smoking, and alcohol consumption on cytokine serum levels. Although the blood samples in this study were collected at the time of first breast cancer diagnosis and prior treatment for women with breast cancer, cancer could influence cytokine expression levels. Hence, the model was adjusted for breast cancer as well. Table 3 presented cytokines with statistically significant (*p* < 0.05) predictive models and coefficients of respective coverable (obesity, T2D, HTN, ethnicity) only. 

The data in Table 3a showed that obesity alone was independently associated with an increase in the expected geometric mean for EGF (+26%), MCP1 (+17%), MIP-1b (+20%), and MDC (+10%), as well as Groα (+45%) and Leptin (+45%). T2D alone was associated with an increase in the expected geometric mean for TGFβ1 (+66%), TGFβ2 (+48%), and Leptin (+62%) independently (Table 3b). HTN was associated with an increase in the expected geometric mean for TNFα (+20%), MIP-1b (+26%), and VEGFα (+32%) (Table 3c). As the data are shown in Table 3d, independent of obesity, T2D, and HTN, AA women were found to increase the geometric mean for MDC (+32%), VEGFα (+48%), and Groα (+15%). In comparison, LA women were more likely to be associated with increases in the expected geometric mean for IP10 (+17%) and MCP1 (+17%). The data can be summarized as obesity-associated cytokines, including EGF, MCP1, MDC, MIP-1b, and Groα. TGFβ1 and TGFβ2 were T2D-associated cytokines, and MIB-1b, TNFα, and VEGFα were HTN-associated cytokines for those AA women and LA women. Leptin was independently associated with both obesity and T2D. In addition, MDC, Groα, and VEGFα were more associated with AA women, and MCP1 and IP10 could be more related to LA women. Other cytokine levels were not significantly associated with comorbidity in this AA and LA women cohort. 

### 3.3. Association of Increasing Obesity-Associated Cytokines and BMI

**Figure 2:**
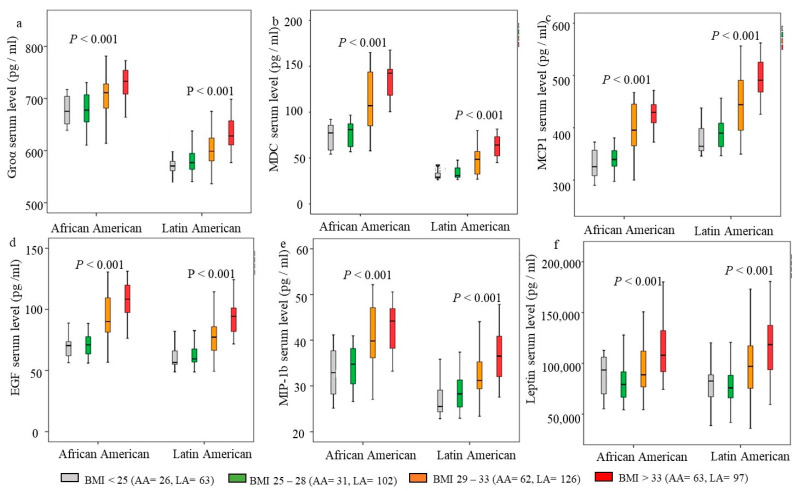
Association of cytokines with body mass index (BMI) in African American (AA) and Latin American (LA) women. The cohort was stratified according to BMI and ethnicity. BMI and ethnicity clustered Boxplot. The predicted levels of cytokines and Leptin from each linear regression model were log (10) transformed, and the geometric median (solid line), interquartile ranges (box), and outliers (vertical line) were presented in each BMI group in AA and LA, respectively. (**a**) Groα serum levels, (**b**) MDC serum level, (**c**) MCP1 serum level, (**d**) EGF serum level, (**e**) MIP-1b serum level, (**f**) Leptin serum level. Kruskal–Wallis one-way ANOVA tests were used to determine the statistical differences; *p* < 0.05 was considered statistical significance.

Since around 37% of AA and LA women in this study were overweight (BMI = 25–29), we stratified the cohort according to BMI to further determine the relationship between BMI and cytokines. Based on the percentiles of BMI in this cohort, we divided BMI into four subgroups: BMI < 25 kg/m^2^ (average body weight), BMI = 25–28 kg/m^2^ (50 percentile), BMI = 29–33 kg/m^2^ (75 percentile), BMI > 33 kg/m^2^. The cohort of women was then stratified into four groups according to their BMI. Notably, several obesity-associated cytokines were associated with specific ethnic groups. Therefore, the cohort was also stratified as AA and LA groups. Figure 2 summarizes the association between increasing cytokine levels and BMI in AA and LA. The increased MCP1 and MIP-1b serum levels occurred at BMI > 25 kg/m^2^ in both AA and LA (Figure 2c,e). Groα, MDC, and EGF levels increased at BMI > 28 kg/m^2^ (Figure 2a,b,d). Comparing the two ethnic groups, the levels of Groα, MDC, EGF, and MIP-1b were significantly higher in AA than in LA (Figure 2a,b,d,e), but the MCP1 level was higher in LA in each BMI group (Figure 2c). Leptin levels were significantly high at BMI > 33 kg/m^2^ in AA, while a significantly high level of Leptin occurred at BMI > 28 kg/m^2^ in LA (Figure 2f). Overall, the data demonstrate an increase in cytokines with increasing BMI, beginning with an overweight BMI > 25 kg/m^2^. The obesity-associated cytokines, Groα, MDC, EGF, and MIP-1b, were significantly higher in AA than in LA. The data confirmed that LA was likelier to have high MCP1 serum levels.

### 3.4. Association of Cytokines and Breast Cancer

Next, we examined the association of serum levels of those cytokines with breast cancer. The cytokines were log10 transformed and included in linear regression models as dependent variables, and breast cancer coded as 1 (cases) and 0 (control) were covariable. Obesity, T2D, HTN, age, and ethnicity were also included in the models as covariable to adjust their influence on cytokine levels. Table 4 summarizes those cytokines significantly associated with breast cancer (both predictive models and coefficients reached statistical significance). As the data are shown in Table 4, breast cancer was associated with an increase in the expected geometric mean for Groα (+8%), MIP-1b (+23%), IP10 (+40%), TNFα (+43%), TGFβ1 (+41%), and TGFβ2 (+41%). Other cytokines were also analyzed, but their levels were not significantly associated with breast cancer. Notably, among the breast cancer-associated cytokines (Table 4), Groα and MIP-1b were also significantly associated with obesity, MIP-1b and TNFα were HTN-associated cytokines, and TGFβ1 and TGFβ2 were T2D-associated cytokines. 

**Figure 3:**
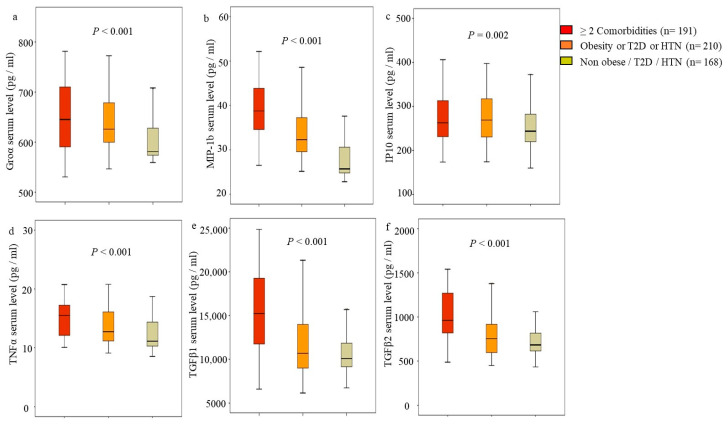
Association of breast cancer-associated cytokines and comorbidities. African American and Latin American women were stratified into three groups according to comorbidities: (1) those who have two or more comorbidities (obesity, type-2 diabetes (T2D), hypertension (HTN), (2) those who have obesity or T2D or HTN (signal condition), and (3) those with nonobesity/T2D/HTN. The predicted levels of the indicated cytokines from the regression model were anti-log (10) transformed, and Boxplots were made. The geometric median (solid line), interquartile ranges (box), and outliers (vertical line) were presented according to the number of comorbidities. (**a**) Predicted Groα serum levels, (**b**) predicted MIP-1 level, (**c**) predicted IP10 level, (**d**) predicted TNFα level, (**e**) predicted TGFβ1 level, (**f**) predicted TGFβ2 level. The significant differences among groups were determined by Kruskal–Wallis one-way ANOVA. *p* < 0.05 was statistically significant.

To understand any synergistic effect for women with more than two comorbidities, the cohort of AA and LA was subgrouped into three groups according to their comorbidities, as follows: those who have two or more comorbidities (obesity, T2D, HTN), those who have obesity or T2D or HTN, and those with non-obesity/T2D/HTN. The data in Figure 3 showed an increase in all breast cancer-associated cytokines in women with obesity, T2D, and HTN compared to non-obese/T2D/HTN. Except for the IP10 level, the predicted levels of Groα, MIP-1b, TNFα, TGFβ1, and TGFβ2 were further increased significantly in women with ≥2 commodities (Figure 3).

### 3.5. Identifying Cytokines’ Levels for Increasing Breast Cancer Risk

Then, we further analyzed the odds ratio (OR) for breast cancer risk by those cytokines in AA and LA women. All cytokines’ serum levels were subgrouped according to their percentile ranges in breast cancer patients: ≤50 percentile, >50 and ≤75 percentiles, and ≥75 percentile, respectively. Women with breast cancer were stratified into three groups according to the level of cytokines. The analysis was performed in different ethnic groups (AA and LA) and different age groups (≤50 years and >50 years). Since those cytokines were associated with comorbidities, Logistic regression with multivariate-adjusted for obesity, T2D, and HTN was used to assess the association of each cytokine with breast cancer. The model was adjusted for age when the analysis was performed in different ethnic groups and for ethnicity when the study was conducted in various age groups. Table 4 summarizes the estimated OR for increased breast cancer risk per cytokine level. The reference level was the respective cytokine’s 50 percentile level. 

The data showed that the high serum levels of Groα (≥708 pg/mL), IP10 (≥389), and TNFα (≥21 pg/mL) increased OR for breast cancer risk significantly in both AA and LA and both ages ≤ 50 years and age > 50 years groups (Table 5). However, the increased OR for breast cancer risk occurred at Groα level ≥ 637 pg/mL in AA (Table 5). The levels of TGFβ1 (≥46,713 pg/mL) and TGFβ2 (between 1005 and 2998 pg/mL) showed increasing O.R.s for breast cancer risk in LA only (Table 5). MIP-1b level did not show a significant increase in OR for breast cancer risk in AA and LA women.

As the data are shown in Table 5, the levels of Groα ≥ 708 pg/mL, MIP-1b ≥ 58 pg/mL, IP10 ≥ 389, TNFα ≥ 21 pg/mL, TGFβ1 ≥ 18456, and TGFβ2 ≥ 1005 pg/mL were increased O.R.s for breast cancer risk significantly in age ≤ 50 years group. In the age > 50 years group, Groα ≥ 637 pg/mL, IP10 ≥ 389, and TNFα ≥ 21 pg/mL were associated with increases in O.R.s (Table 5). TGFβ2 levels between 1005 pg/mL and −2998 pg/mL increased OR significantly. However, the significance disappeared in the highest-level group (TGFβ2 ≥ 2999 pg/mL). MIP-1b level was not increasing OR in the age >50 years group (Table 5).

In addition to these cytokines, MDC level ≥ 1271 pg/mL (OR = 1.9, 95% CI: 1.0–3.6, *p* = 0.04) and G-CSF ≥ 47.4 pg/mL (OR = 2.3, 95% CI: 1.0–5.0, *p* = 0.04) were significantly associated with increased breast cancer risk in the age > 50 years group only. We did not find an association of other cytokines’ levels with breast cancer risk in this cohort of women.

## 4. Discussion

Cytokines are critical mediators that regulate immune and inflammatory responses. Their effects are mediated through complex regulatory networks. Human cytokine profiles could define patient subgroups and represent new potential biomarkers for many diseases, including cancer. Chronic inflammation from comorbidities could activate cytokines and trigger cellular events, promoting malignant transformation of cells and carcinogenesis [35].

Vulnerable populations in South Los Angeles, such as AA and LA women, have a high incidence of comorbidities (obesity, T2D, HTN) that elevate the risk of comorbidity-driven breast cancer [36,37]. Understanding comorbidities associated with cytokines expression will help to facilitate efficient intervention and treatment strategies for breast cancer prevention. In this study, we analyzed 570 AA and LA women with and without breast cancer and comorbidities and found that obesity, T2D, and HTN were independently associated with a group of cytokines. We also found differentiated cytokines panels between AA and LA women from South Los Angeles communities. Our study is the first time that two or more comorbidities further increase specific cytokine levels, potentially enhancing breast cancer risk, especially for AA and LA women at age < 50 years. 

Adipose tissue remodeling during obesity provides a plethora of intrinsic and extrinsic signals capable of triggering an inflammatory response and activation of cell signings, such as the JNK and NF-kB signaling pathways and the target genes, e.g., IL-6, TNFα, interferons, and MCP-1 [38]. Studies in obese individuals also reported the association of obesity with IFN-γ, TNF-α, and interleukin proteins [26,27,39]. Few groups studied obesity-induced inflammatory and cytokine profile changes in AA [27,28], and no study was focused on LA. Denis et al. evaluated cytokines’ profile from 39 obese African American women with and without T2D and found interleukin-4, soluble CD40 ligand, and chemokine (C-C motif) ligand 3 (CCL3) were independent of T2D associated with obese African American women [28]. A study from Williams et al. was on AA women with obesity and elevated HbA1c. The uniqueness of our research was focused on AA and LA women and compared cytokine profiles in obese and non-obese women with and without comorbidities. Our data showed that EGF and chemokines Groα/CXCL1 and CC chemokine ligands, CCL2 (MCP-1), CCL4 (MIP-1b), and CCL22 (MDC) were independent of T2D and HTN associated with obese AA and LA women. Adjusted for age, smoking, alcohol consumption, and other comorbidities, the predicted serum levels of these chemokines were found to be increased in women who were overweight. The levels of EGF, Groα, and MDC were higher in AA than in LA women, while the level of MCP1 was higher in LA women compared to AA women (Figure 2). Among those chemokines, Groα/CXCL1 was predicted to cause a 45% increase in obesity. The epigenetic mechanism may be associated with the rise in Groα/CXCL1 in obese individuals. Ali et al. found 28 proinflammatory genes, including Groα/CXCL1, were significantly hypomethylated in obese individuals compared to lean controls [40]. Elevated MCP-1/CCL2 was reported in obese adults and obese Mexican American children [41,42,43], and the mechanism included obesity-inducing inflammatory and triggering NF-kB signaling [38]. 

Interleukin proteins, IL6 and IL8, were reported to be upregulated in obese individuals by several previous studies [26,44]. The elevation of circulating IL6 and IL8 levels in obese individuals may be more likely related to T2D and insulin resistance [28,45]. However, we did not observe differences in serum levels of IL-6 and IL8 in this cohort of obese and non-obese women with and without T2D. In addition to IL6 and IL8, clinical studies revealed the association between TGF-β1 and nephropathy in T2D [46,47]. Consistent with those clinical studies, we identified that transforming growth factor family members TGF-β1 and TGF-β2 were associated with T2D independent of obesity and other comorbidities. After adjusting for obesity, comorbidities, age, smoking, alcohol consumption, and ethnicity, our model predicted 66% and 48% increases of TGFβ1 and TGFβ2 by T2D, respectively, in this cohort of AA and LA. It may suggest different patterns of cytokine expression from this cohort of AA and LA.

Furthermore, our study found Leptin was independently associated with obesity and T2D in AA and LA women. Leptin is the product of the obese gene [48]. There are various signal transduction pathways, such as the Jak/STAT3, MAPK, and PI3K pathways could collectively regulate the leptin’s metabolic effects [49]. Leptin was identified to be independently associated with obesity and T2D in this study. Leptin was initially considered for use in treating obesity; however, its altered expression and receptor expression led to Leptin resistance in obesity-related complications [50]. More mechanisms studies in understanding the pathogenesis of obesity-related disorders and their role will provide new alternatives in obesity and obesity-associated metabolic disease treatment. 

Increased blood pressure may be a stimulus for inflammation, a possible mechanism underlying the well-established role of hypertension [51]. An early study by DeLoach et al. examined 484 AA, including people with and without obesity and with and without HTN. Plasma C-reactive protein, IL-6, Plasminogen activator inhibitor 1, TNF-α, TNF-αR, and adiponectin were analyzed in their study. Only TNF-α was found to be associated with HTN [29]. Another cross-sectional study involving 508 healthy men analyzed the association between blood pressure and baseline plasma concentrations of two inflammatory markers: intercellular adhesion molecule-1 (sICAM-1) and IL-6. A significant association of plasma IL-6 with systolic and diastolic blood pressure levels was seen in their study [52]. We included African AA and LA women with and without obesity and with or without HTN in the survey. We also included women with and without T2D and measured a penal of 19 cytokines. TNFα, not IL-6, was identified to be significantly associated with HTN in our study. We observed increasing serum levels of Groα/CXCL1, MIP-1b/CCL4, TNFα, VEGFα, and TGFβ1 in women with HTN. However, after adjusting for other comorbidities (obesity, T2D, and breast cancer), age, smoking, alcohol consumption, and ethnicity, MIP-1b/CCL4, TNFα, and VEGFα remained independently associated with HTN in our study cohort. 

A population-based study by Stowe et al. showed that cytokine levels were influenced by ethnicity [53]. The study demonstrated that IL-1ra, IL-6, and C-reactive protein levels were influenced by ethnicity. Furthermore, their study found inflammatory profiles for Mexican Americans were lower than for non-Hispanic whites and non-Hispanic blacks [53]. We also compared the expression of 19 cytokines in AA and LA women. In agreement with Stowe’s study, we observed serum levels of Groα/CXCL1, MIP-1b/CCL4, MDC/CCL22, EGF, and VEGFα were significantly lower in LA women than in AA women. While, after adjusting for comorbidities (obesity, T2D, and HTN), breast cancer, age, smoking, and alcohol consumption, MDC/CCL22, VEGFα, and Groα remained significantly associated with AA. The expected geometric mean for MDC/CCL2 was increased by 32%, VEGFα was increased by 48%, and Groα/CXCL1 was increased by 15% in AA compared with LA. However, MCP-1/CCL2 and IP-10/CXCL10 were significantly higher in LA than in AA. The expected geometric mean for MCP-1/CXCL2 and IP10/CXCL10 increased by 17% in LA compared to AA. 

The obesity–inflammation axis regulates metabolic syndrome that might underlie many of the associated risks with cancer. Substantial changes occur within the adipose tissue microenvironment during the development of obesity due to a combination of adipogenesis and lipogenesis, processes regulated by insulin/insulin resistance signaling [8,54]. These changes could increase the production and release of numerous cytokines into the immune cell landscape’s microenvironment remodeling and promote tumor microenvironment development [8,54]. The cytokine profiles are explicitly associated with obesity, T2D, and HTN and might lead to an increased cancer risk. We had the opportunity in this study to assess the association of comorbidities (obesity, T2D, and HTN)-associated cytokine profiles with the risk of breast cancer and identify breast cancer risk-associated cytokine profiles in AA and LA women. We found that Groα/CXCL1 (obesity-associated), MIP-1b/CCL4 (obesity- and HTN-associated), TNFα (HTN-associated), and TGFβ1 and β2 (T2D-associated) were also significantly associated with breast cancer independently. When comorbidities present together, i.e., obesity, T2D, and HTN, any two in combination had additive associations between breast cancer and those cytokines. This suggests that (1) increasing the serum levels of these cytokines alone is associated with breast cancer, and (2) any one or two comorbidities enhanced the association of cytokines with breast cancer.

TNFα was predicted to have a 48% increase in breast cancer compared to no cancer in our model. TNF-α is an essential pro-inflammatory cytokine secreted by stromal cells, tumor-associated macrophages, and cancer cells [55]. It was highly expressed in tumor cells of biopsies from most breast cancer patients [56]. TNF-α stimulates the stromal cells and releases elevated levels of CCL2, CXCL8, and CCL5, which have tumor-promoting solid activities in general and breast cancer, mainly when derived from stroma cells [57]. Furthermore, Zhang et al. reported that CXCL1 can mediate obesity-associated adipose stromal cell trafficking and functions in the tumor microenvironment, promoting prostate cancer progression [58]. Our previous study’s in vitro cell model showed higher Groα/CXCL1 expression in breast cancer cells, especially in TNBC cells, than in non-cancer cells [59]. A high expression of Groα/CXCL1 in breast cancer promoted cell invasion via the MAPK pathway [59]. Groα/CXCL1 was identified in this study as obesity-associated chemokines, and it was also associated with breast cancer independent of obesity. With comorbidities, the level of Groα/CXCL1 could be further increased significantly, which could argue the further increased risk of breast cancer. The data from this study showed that high serum levels of TNFα and Groα/CXCL1 increased OR for breast cancer at age ≤ 50 and age > 50 years in both AA and LA women. It is well known that obesity is associated with postmenopausal breast cancer and may be more related to ER-positive breast cancer [4,5]. However, several studies showed obesity may also be associated with increasing premenopausal breast cancer risk and could be related to both ER-positive and ER-negative breast cancer [22,60,61]. The median ages of having breast cancer were 49 years for LA and 51 years for AA in our study. The comorbidity-driven inflammation and increasing cytokines, such as TNFα and Groα/CXCL1, might contribute to the early onset of breast cancer in this cohort of AA and LA women.

IP-10/CXCL10 is a pro-tumorigenic chemokine and is secreted mainly by malignant rather than non-malignant tissues, correlated with the progression of breast cancer [31]. In this study, we found that IP10 was significantly associated with breast cancer independent of comorbidities and predicting breast cancer risk in both AA and LA women in age ≤ 50 years and age > 50 years groups. 

The association of MIP-1b/CCL4, TGFβ1, and TGFβ2 and breast cancer were complicated. TGFβ is known to play a tumor suppressor role at an early stage of breast cancer and promote cancer progression in the late stage of breast cancer [62]. The connection of MIP-1b/CCL4 with breast cancer was also more likely to promote tumor progression. The CCL4-CCR5 axis contributed to breast cancer metastasis to the bone by mediating the interaction between cancer cells and fibroblasts in the bone cavity [63]. Our study identified serum levels of MIP-1b/CCL4, TGFβ1, and TGFβ2 were independently associated with breast cancer. Comorbidity-driven inflammation and increasing MIP-1b/CCL4 serum levels and TGFβ1 and β2 increased the association. Furthermore, increasing serum levels of MIP-1b/CCL4 and TGFβ1 and β2 increased OR for breast cancer at the age ≤ 50 years group. TGFβ1 and TGFβ2 were also more likely to increase OR for breast cancer in LA women. 

This study is limited because only AA and LA were examined. Therefore, the results could not be compared with other ethnic groups. Additionally, the cross-sectional design allows us to demonstrate the associations and draw conclusions related to cause and effect. However, since the comorbidities were occurring before breast cancer diagnosis, therefore, we can conclude that comorbidity-induced inflammation upregulated a specific panel of cytokines that might increase breast cancer risk for AA and LA women. Both AA and LA are more likely to have comorbidities and be diagnosed with breast cancer at a younger age. It is known that the frequency of TNBC was higher in younger age AA with breast cancer, which led to poor breast cancer survival. In this study, we found that obesity, T2D, HTN, and breast cancer were each independently associated with an increase in a panel of inflammatory cytokines and further increases in those cytokines when more conditions were present that might be additive to the risk for breast cancer. There is a need for strategic, community-oriented, and culturally appropriate public health interventions to reduce obesity and comorbidity-induced inflammation. The specific panel of cytokines identified in this study could be biomarkers for designing intervention strategies, such as increased physical activity and lifestyle changes, and be used to assess the intervention strategies’ effectiveness. 

## 5. Conclusions

Our data showed that comorbidity-induced inflammation upregulated a specific panel of cytokines that might increase breast cancer risk for AA and LA women.

## Figures and Tables

**Table 1:** Study population.

Characteristics	Total = 570
	*n* (%)
Ethnicity	
African American	182 (31.9)
Latin American	388 (68.1)
Age	
30–40	117 (20.5)
41–50	208 (36.5)
51–60	180 (31.6)
>60	65 (11.4)
Cancer	
Breast cancer	261 (45.8)
Non-Cancers	309 (54.2)
Body Mass Index (BMI)	
Obese (≥30)	261 (45.8)
Overweight (>25 and <30)	210 (36.8)
Normal Weight (≤25)	99 (17.4)
Type-2 Diabetes (T2D)	
Yes	160 (28.1)
No	410 (71.9)
Hypertension (HTN)	
Yes	192 (33.7)
No	378 (66.3)

**Table 2:** Serum levels of cytokines according to ethnicity, body mass index (BMI), type-2 diabetes (T2D), and hypertension (HTN).

Cytokines		Median Level (pg/mL)										
		Ethnicity		BMI			Breast Cancer		T2D		HTN	
	Total	AA ^a^	LA ^aa^	≥30	25–29	<25	Cases	Control ^b^	Yes	No	Yes	No
EGF	86.9	**119.8 ^**	79.4	**95.2 ^^**	72.5	86.8	**102.6 ^^**	77.4	73.7	87.3	92.8	78.8
FGF2	44.1	38.9	47.0	41.0	47.4	44.2	42.8	45.1	41.1	43.8	37.8	47.4
G-CSF	24.8	25.3	24.5	24.0	24.6	26.8	**27.2 ^**	22.4	26.1	23.8	25.3	24.2
GM-CSF	11.2	12.7	11.0	12.0	11.0	11.5	9.1	12.8	10.3	11.7	9.1	12.3
Fractalkine	48.3	45.7	49.4	47.3	49.2	49.1	44.9	51.1	44.7	49.2	47.6	45.9
INFα2	15.6	14.0	17.1	16.0	13.5	18.3	14.9	17.9	14.0	16.0	17.0	13.8
IFNɣ	7.4	7.3	7.4	6.5	8.0	7.8	7.4	7.4	4.8	7.9	6.8	7.9
TNFα	13.9	14.7	13.7	15.3	14.3	11.8	**17.3 ^^**	12.2	**15.7 ^**	13.2	**16.7 ^^**	12.4
Groα	636.1	**682.1 ^^**	615.7	**644.5 ^^**	626.5	618.5	**657.1 ^^**	610.0	623.5	642.3	**654.6 ^**	627.7
IP10	248.5	240.4	254.2	270.0	248.0	219.2	**300.6 ^^**	225.1	259.3	243.6	261.7	236.8
MCP1	428.0	369.0	448.5	**484.3 ^^**	377.2	380.1	447.7	405.0	**467.0 ^**	404.2	463.8	402.2
MIP-1b	34.9	**42.0 ^^**	32.6	**41.0 ^^**	32.6	25.5	**41.0 ^^**	29.3	40.5	32.3	**44.1 ^^**	29.5
MDC	866.9	**1045 ^^**	762	**974.4 ^^**	782.9	659.2	**938.2 ^^**	755.9	904.1	862.3	**939.5 ^^**	830.4
IL6	19.2	16.2	20.3	14.4	18.5	28.5	16.2	23.1	14.0	22.2	17.1	19.4
IL8	11.6	11.3	11.7	12.8	10.1	10.9	13.2 ^	9.6	11.7	11.5	15.1 ^^	9.2
VEGFα	111.4	**152.9 ^^**	97.6	**136.3 ^^**	86.1	78.3	**126.4 ^^**	97.8	109.3	109.7	**131.9 ^^**	100.0
TGFβ1	17,979.0	19,610.5	17,707.0	19,745.0	13,358.5	20,362.0	**25,667.0 ^**	9123.0	**32,977.8 ^^**	29,411.8	20,066.0	18,152.0
TGFβ2	993.7	1219.0	892.8	958.7	906.2	1105.5	**1508.0 ^**	652.2	**2063.2 ^**	1832.2	1275.0	917.6
Leptin	91,456.0	92,404.0	90,721.0	**110,757.0 ^^**	85,119.0	74,637.0	96,702.0	84,184.0	**185,837.5 ^^**	141,197.2	94,162.0	110,341.0

^ *p* ≤ 0.05 comparing AA. vs. LA, BMI ≥ 30 vs. >25 and ≤30 vs. ≤30, T2D vs. non-T2D, HTN vs. non-HTN; ^^ *p* ≤ 0.01 comparing AA. vs. LA, BMI ≥ 30 vs. > 25 and ≤30 vs. ≤30; ^a^ AA.: African American, ^aa^ Latin American; ^b^ Control: women without cancers.

**Table 3:** Linear regression with multivariate analysis estimated the association of cytokines with obesity, T2D, HTN, and ethnicity.

a. Association of cytokines and obesity				
Outcome	Coefficients			Expected geometric mean ratios (95% CI) by obesity (Yes vs. No)
Cytokines	B (Obesity)	Std. Error	*p*	
EGF ^	0.16	0.05	0.001	1.26 (1.0, 1.8)
MCP1 ^	0.10	0.03	<0.001	1.17 (1.1, 1.45)
MIP-1b ^	0.07	0.03	0.04	1.20 (1.0, 1.38)
MDC ^	0.08	0.03	0.004	1.10 (1.06, 1.38)
Groα ^	0.04	0.01	0.001	1.45 (1.03, 1.13)
Leptin ^	0.16	0.05	0.001	1.45 (1.15, 1.78)
b. Association of cytokines and T2D				
Outcome	Coefficients			Expected geometric mean ratios (95% CI) by T2D (Yes vs. No)
Cytokines	B (T2D)	Std. Error	*p*	
TGFβ1 ^	0.22	0.08	0.04	1.66 (1.17, 2.29)
TGFβ2 ^	0.17	0.07	0.04	1.48 (1.10, 2.04)
Leptin ^	0.21	0.07	<0.001	1.62 (1.26, 2.0)
c. Association of cytokines and HTN				
Outcome	Coefficients			Expected geometric mean ratios (95% CI) by HTN (Yes vs. No)
Cytokines	B (HTN)	Std. Error	*p*	
TNFα ^^	0.08	0.03	0.01	1.20 (1.05, 1.4)
MIP-1b ^^	0.10	0.04	0.04	1.26 (1.10, 1.5)
VEGFα ^^	0.12	0.06	0.02	1.32 (1.02, 1.7)
d. Association of cytokines and ethnicity				
Outcome	Coefficients			Expected geometric mean ratios (95% CI) by ethnicity (AA.* vs. LA **)
Cytokines	B (AA *)	Std. Error	*p*	
MDC ^	0.12	0.03	<0.001	1.32 (1.15, 1.55)
IP10 ^	−0.08	0.05	0.02	0.83 (0.71, 0.74)
MCP1 ^	−0.08	0.03	0.01	0.83 (0.72, 0.96)
VEGFα ^	0.17	0.06	0.007	1.48 (1.12, 2.0)
Groα ^	0.06	0.01	<0.001	1.15 (1.1, 2.2)

The indicated cytokines as the dependent variable were log (10) transformed to correct for skewness in regression models. Obesity, T2D, HTN, breast cancer, and ethnicity were all included in the model as co-variables. In addition, the modes were also adjusted for age, smoking, and alcohol consumption. Those models and the coefficients that achieved statistical significance were presented in the Table (^ model’s *p* ≤ 0.001, ^^ model’s *p* ≤ 0.01). The coefficient results were also anti-log transformed and presented as geometric mean ratios representing multiplicative increases in the dependent variables (cytokines). CI, confidence interval. * AA, African American; ** LA, Latin American.

**Table 4:** Liner regression analyzing the association of various cytokines with breast cancer.

Outcome	Coefficients			Expected Geometric Mean Ratios (95% CI) by Breast Cancer (Yes vs. No)
Cytokines	B (Cancer)	Std. Error	*p*	
Groα	0.03	0.01	0.002	1.08 (1.03, 1.13)
MIP-1b	0.09	0.03	0.006	1.23 (1.06, 1.44)
IP10	0.15	0.03	<0.001	1.40 (1.23, 1.58)
TNFα	0.15	0.03	<0.001	1.43 (1.23, 1.62)
TGFβ1	0.15	0.07	0.029	1.41 (1.03, 1.91)
TGFβ2	0.15	0.06	0.018	1.41 (1.07, 1.91)

Models were adjusted for obesity, T2D, HTN, age, and ethnicity.

**Table 5:** Logistic regression with multivariate analysis estimated the association of cytokines and breast cancer.

Cytokines (pg/mL)	African American		Latin American		Age ≤ 50		Age > 50	
	OR ^ (95% CI)	*p*	OR ^ (95% CI)	*p*	OR * (95% CI)	*p*	OR * (95% CI)	*p*
**Groα**								
≤636	1		1		1		1	
637–707	4.2 (1.3–13.3)	**0.015**	1.7 (0.9–3.3)	0.123	1.9 (0.9–4.2)	0.094	2.3 (1.0–5.3)	**0.04**
≥708	8.7 (2.9–25.6)	**<0.001**	5.4 (1.7–17.6)	**0.005**	4.0 (1.4–11.0)	**0.009**	7.0 (2.6–19.3)	**<0.001**
**MIP-1b**								
≤35	1		1		1		1	
36–57	1.9 (0.8–4.9)	0.155	1.2 (0.7–2.1)	0.463	1.4 (0.7–2.4)	0.32	1.6 (0.8–3.4)	0.194
≥58	1.1 (0.5–2.3)	0.828	1.6 (0.9–2.9)	0.086	1.8 (1.0 -3.3)	**0.04**	1.1 (0.6–2.3)	0.744
**IP10**								
≤250	1		1		1		1	
251–388	1.5 (0.6–3.4)	0.35	1.5 (0.9–2.6)	0.136	1.1 (0.5–2.1)	0.81	1.6 (0.8–3.2)	0.21
≥389	5.1 (2.0–13.3)	**0.001**	3.0 (1.7–5.1)	**<0.001**	3.6 (1.8–6.9)	**<0.001**	3.2 (1.4–7.2)	**0.004**
**TNFα**								
≤14	1		1		1		1	
15–20	1.5 (0.6–2.8)	0.533	1.6 (0.9–2.8)	0.105	1.4 (0.7–2.4)	0.17	1.4 (0.6–3.0)	0.46
≥21	2.7 (1.0–6.9)	**0.041**	3.1 (1.8–5.3)	**<0.001**	1.8 (1.0–3.3)	**0.026**	4.1 (2.0–8.6)	**<0.001**
**TGFβ1**								
≤184,55	1		1		1		1	
18,456–46,712	1.0 (0.4–2.3)	0.944	1.9 (1.1–3.2)	**0.026**	2.3 (1.3–4.3)	**0.006**	1.0 (0.4–2.1)	0.924
≥46,713	1.2 (0.5–2.9)	0.698	1.7 (1.0–3.0)	**0.05**	1.8 (1.0–3.4)	**0.047**	1.6 (0.7–3.3)	0.242
**TGFβ2**								
≤1004	1		1		1		1	
1005–2998	2.0 (0.7–5.4)	0.166	2.7 (1.6–4.7)	**<0.001**	2.7 (1.5–5.1)	**0.001**	2.4 (1.1–5.2)	**0.037**
≥2999	1.2 (0.5–2.7)	0.733	1.7 (0.9–2.9)	0.085	1.9 (1.0–3.5)	**0.048**	1.5 (0.7–3.2)	0.339

OR: odds ratio, determined by Logistic regression with multivariate analysis; ^ adjusted for obesity, T2D, HTN, and age; * adjusted for obesity, T2D, HTN, and ethnicity; *p* < 0.05 was statistically significant. Bolded numbers imply significance.

## Data Availability

The data presented in this study are available upon request from the corresponding author and principal investigator, though they are restricted to investigators based in academic institutions.
